# Cigarette smoke increases susceptibility of alveolar macrophages to SARS-CoV-2 infection through inducing reactive oxygen species-upregulated angiotensin-converting enzyme 2 expression

**DOI:** 10.1038/s41598-023-34785-6

**Published:** 2023-05-16

**Authors:** Chin-Wei Kuo, Po-Lan Su, Tang-Hsiu Huang, Chien-Chung Lin, Chian-Wei Chen, Jeng-Shiuan Tsai, Xin-Min Liao, Tzu-Yi Chan, Chi-Chang Shieh

**Affiliations:** 1 Institute of Clinical Medicine, College of Medicine, National Cheng Kung University, 138 Sheng-Li Road, Tainan, 70403 Taiwan; 2 Division of Chest Medicine, Department of Internal Medicine, National Cheng Kung University Hospital, College of Medicine, National Cheng Kung University, Tainan, Taiwan; 3 Department of Pediatrics, National Cheng Kung University Hospital, College of Medicine, National Cheng Kung University, Tainan, Taiwan

**Keywords:** Infectious diseases, Viral infection, Infectious diseases, Innate immune cells

## Abstract

Alveolar macrophages (AMs) are the drivers of pulmonary cytokine storm in severe acute respiratory syndrome coronavirus 2 (SARS-CoV-2) infection. This study aimed to investigate clinical–regulatory factors for the entrance protein of SARS-CoV-2, angiotensin–converting enzyme 2 (ACE2) in AMs. Human AMs were collected from 56 patients using bronchoalveolar lavage. ACE2 expression in AMs was positively correlated with smoking pack-year (Spearman’s r = 0.347, P = 0.038). In multivariate analysis, current smoking was associated with increased ACE2 in AMs (β-coefficient: 0.791, 95% CI 0.019–1.562, P = 0.045). In vitro study, ex-vivo human AMs with higher ACE2 were more susceptible to SARS-CoV-2 pseudovirus (CoV-2 PsV). Treating human AMs using cigarette smoking extract (CSE) increases the ACE2 and susceptibility to CoV-2 PsV. CSE did not significantly increase the ACE2 in AMs of reactive oxygen species (ROS) deficient Cybb^–/–^ mice; however, exogenous ROS increased the ACE2 in Cybb^–/–^ AMs. N-acetylcysteine (NAC) decreases ACE2 by suppressing intracellular ROS in human AMs. In conclusion, cigarette smoking increases the susceptibility to SARS-CoV-2 by increasing ROS–induced ACE2 expression of AMs. Further investigation into the preventive effect of NAC on the pulmonary complications of COVID-19 is required.

## Introduction

Coronavirus disease 2019 (COVID-19) is an pandemic disease caused by severe acute respiratory syndrome coronavirus 2 (SARS-CoV-2) infection^1^. SARS-CoV-2 infection often leads to acute respiratory distress syndrome (ARDS), and despite intensive care and advanced ventilation support, its mortality rate remains high^2^. Although new generation vaccines are available, the emergence of the SARS-CoV-2 omicron variant poses new challenges due to neutralization escapes^3^. Effective treatment for SARS-CoV-2 infection includes antiviral drugs and immune modulators; however, the high prevalence of infection still results in a large number of COVID-19 deaths^4^. Additionally, the use of immune modulators is associated with an increased risk of secondary infections^4^.

Angiotensin-converting enzyme-2 (ACE2) is a type I integral membrane carboxypeptidase protein and a homolog of human ACE^5^. It is well-known as the entry protein for SARS-CoV-2^6^. The spike protein of the omicron variant has been shown to have increased antibody evasion; however, it still exhibits strong interaction with ACE2^7^. ACE2 is expressed throughout the respiratory system^8^. Transcriptomic and proteomic studies suggest a causal role of ACE2 in the susceptibility to SARS-CoV-2 and disease severity^9^. Many factors, including age, sex, smoking, and chronic obstructive pulmonary disease (COPD), can affect ACE2 expression in the respiratory system^8,10,11^. However, there are only a few studies on the modulators of ACE2 expression in pulmonary immune cells, and most studies investigate ACE2 expression in pulmonary immune cells at the messenger ribonucleic acid (mRNA) level^8,12,13^. Only a few studies have investigated ACE2 expression at the protein level using immunochemical staining^10,14,15^.

Alveolar macrophages (AMs) are the most abundant and frontline immune cells in the lungs, and they are also the major determinative immune cells for early response to respiratory virus invasion^16^. AMs potentially play a key role in coordinating inflammation, damage, and repair procedures that determine the various pathological stages of ARDS^17^. Transcriptional programs of pro–inflammatory genes in AMs are also associated with the clinical outcome of ARDS^18^. For COVID-19, M1 polarization of AMs facilitates SARS-CoV-2 infection^19^. Autopsy and pathological studies of COVID–19 patients indicate that AMs are the drivers of the cytokine storm in SARS-CoV-2 infection^20,21^. While AMs play an important role in severe SARS-CoV-2 infection, studies on regulating ACE2 expression in AMs are still scarce.

Therefore, this study aimed to explore clinical factors for ACE2 expression in AMs. Flow cytometry was used to investigate ACE2 expression in pulmonary immune cells retrieved from bronchoalveolar lavage (BAL) of clinical patients. We also conducted an in vitro study to investigate the relationship between ACE2 expression, cigarette smoke, SARS-CoV-2 infection of AMs, and the role of reactive oxygen species (ROS) on ACE2 expression of AMs.

## Results

### ACE2–expressing AMs are more susceptible to the SARS-CoV-2 pseudovirus

**Figure 1:**
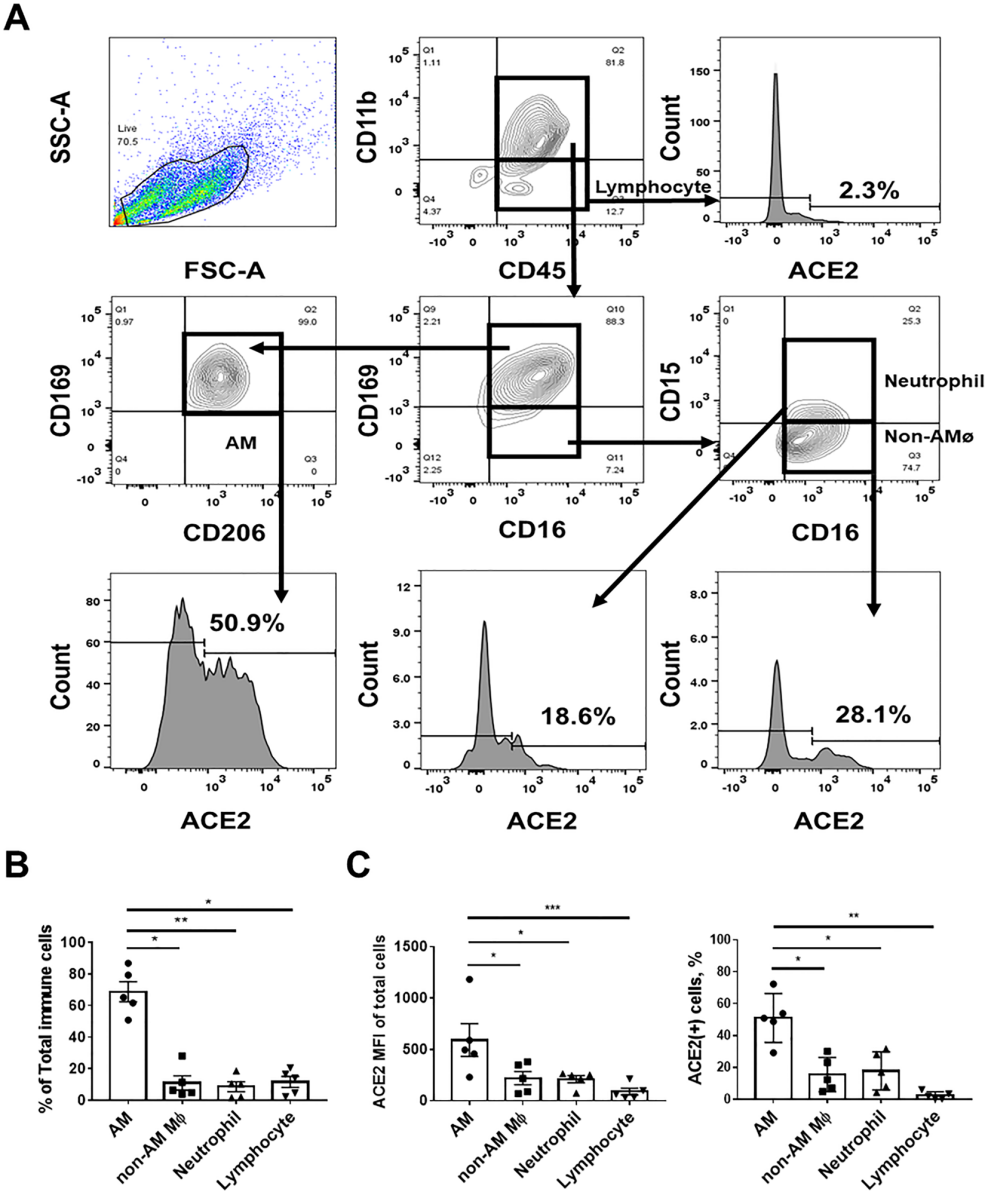
ACE2 expression in BAL-derived pulmonary immune cells. (**A**) Flow cytometry panel of BAL-derived cells. The CD11b^+^CD16^+^CD45^+^CD169^+^CD206^+^ cells were AMs, the CD11b^+^CD15^–^CD16^+^CD45^+^CD169^–^ cells were non-AMs macrophages, the CD11b^+^CD15^+^CD16^+^CD45^+^CD169^–^ cells were neutrophils, and the CD11b^–^CD45^+^ cells were lymphocytes. (**B**) Composition of BAL-derived pulmonary immune cells of non–smoking participants. (**C**) Comparison of ACE2 expression in different pulmonary immune cells. The columns and error bars represent the means and SEM of experiments. The statistically significant differences between groups are indicated with *, **, and *** (*P < 0.05, **P < 0.01, ***P < 0.005, paired *t* test).

**Figure 2:**
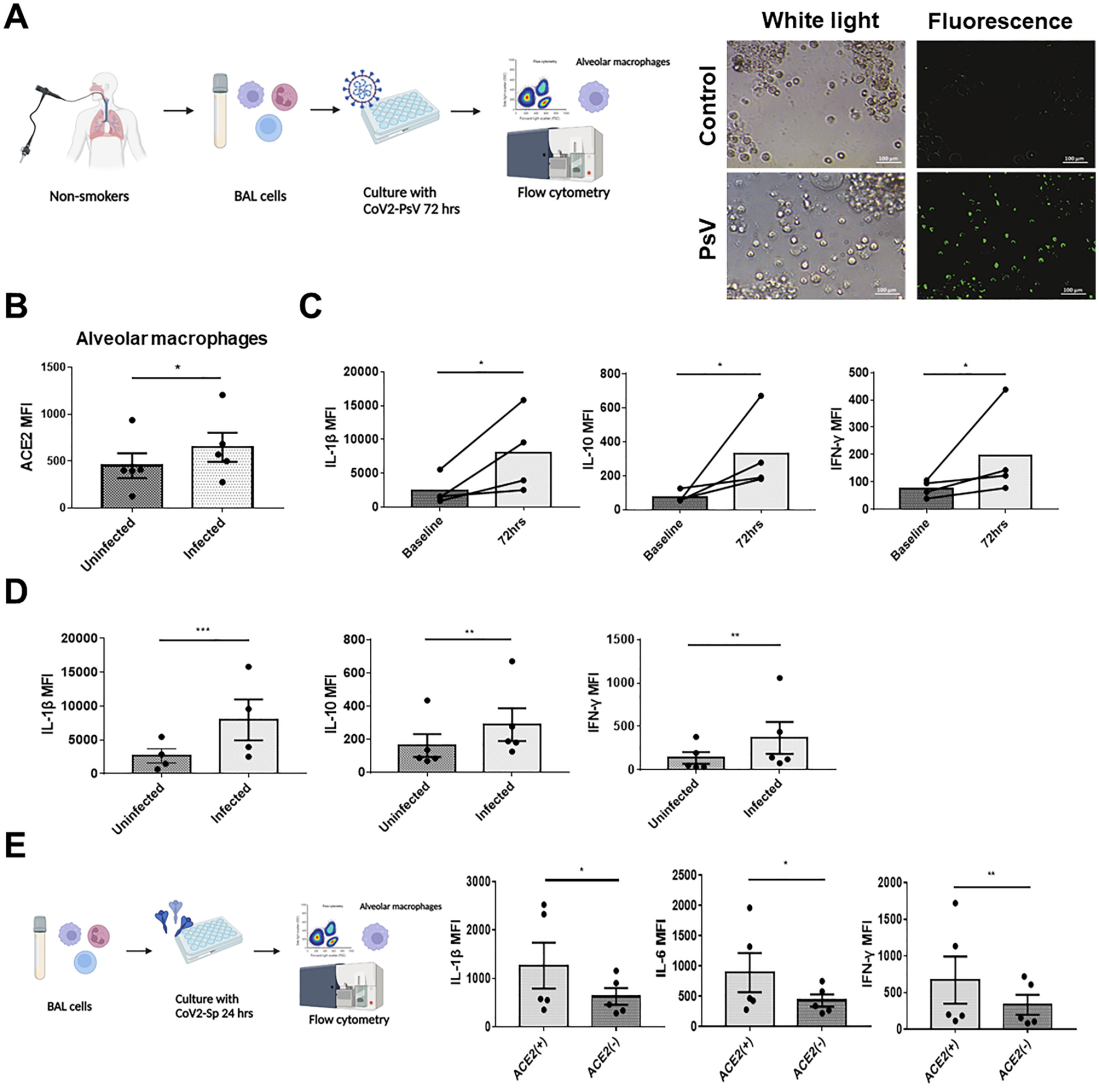
The relationship between ACE2 expression in AMs and susceptibility to SARS-CoV-2 infection. (**A**) BAL-derived cells of non-smokers were treated with 6 μL/well of CoV-2 PsV for 72 h. The infected cells showed green fluorescence. ACE2, IL-1β, IL-10, IFN-γ and CoV-2 PsV of AMs were measured using flow cytometry. (**B**) The comparison of ACE2 of between CoV-2 PsV infected and non-infected AMs. (**C**) The comparison of IL-1β, IL-10, and IFN-γ between AMs before and 72 h after CoV-2 PsV inoculation. (**D**) Comparison of IL-1β, IL-10, and IFN-γ between CoV2-PsV-infected AMs and uninfected AMs. (**E**) BAL-derived cells of non-smokers were incubated with 5 μg/ml of CoV-2 Sp for 24 h, and the ACE2, IL-1β, IL-6, and IFN-γ MFI of AMs were measured using flow cytometry. The columns and error bars represent the means and SEMs of experiments. The statistically significant differences between groups are indicated with *, ** and *** (*P < 0.05, **P < 0.01, ***P < 0.005, paired *t* test).

The expression of ACE2 in human pulmonary immune cells is not well understood. To investigate ACE2 expression in different pulmonary immune cells, we collected pulmonary immune cells using BAL from clinical participants. The gating strategy for immune cells and the flow cytometry panel are shown in Fig. 1A. According to BAL, AMs were the most abundant immune cells (Fig. 1B), consistent with a previous study^22^, and they expressed more ACE2 than other pulmonary immune cells (Fig. 1C). As ACE2 is known to be the entry protein for SARS-CoV-2^6^, we further investigated the relationship between ACE2 expression and susceptibility to CoV-2 PsV infection, as well as the subsequent cytokine production of human AMs (Fig. 2A). After incubating AMs with CoV-2 PsV for 72 h, CoV-2 PsV-infected AMs had higher ACE2 expression **(**Fig. 2B) and significantly increased production of IL-1β, IL–10, and IFN–γ (Fig. 2C). CoV-2 PsV-infected AMs also had significantly higher IL-1β, IL-10, and IFN-γ production than uninfected AMs (Fig. 2D). We then treated AMs with CoV-2 Sp, and we observed that ACE2-expressing AMs produced higher levels of IL-1β, IL-6, and IFN-γ compared to those without ACE2 expression (Fig. 2E). These results suggest that AMs are the majority of immune cells in the lung, with relatively high ACE2 expression. Therefore, AMs with higher ACE2 expression are more susceptible to SARS-CoV-2 infection and produce more inflammatory cytokines upon infection.

### Cigarette smoking is associated with increased ACE2 expression in AMs

**Figure 3:**
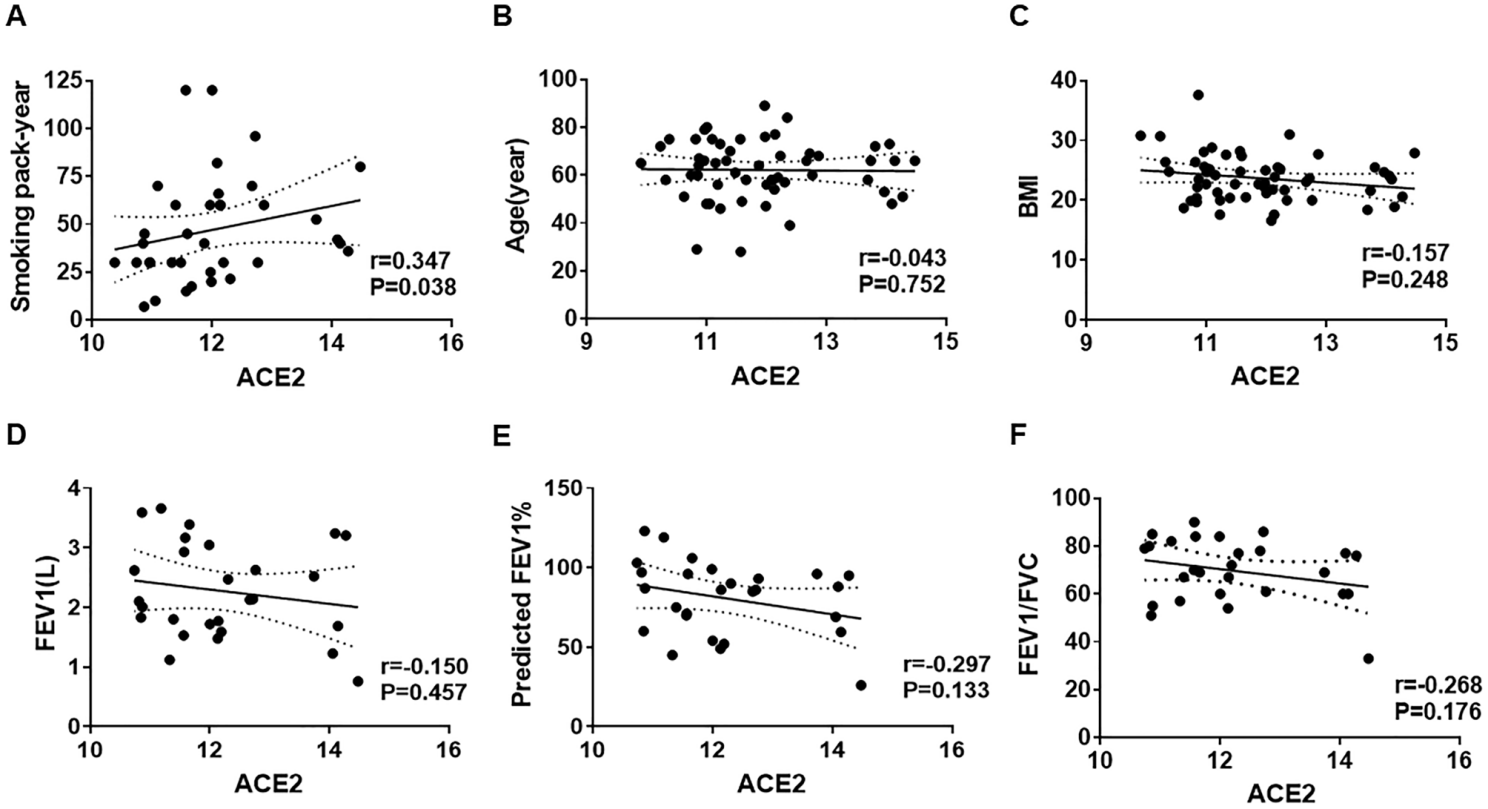
Correlation between ACE2 expression in AMs and clinical factors. (**A**) Smoking pack-year (n = 36), (**B**) Age (n = 56), (**C**) BMI (n = 56), (**D**) FEV1 (n = 27), (**E**) measured FEV1/predicted FEV1 (n = 27) and (**F**) FEV1/FVC (n = 27). ACE2 MFI was log_2_ transformed. Correlation was determined using Spearman’s correlation test.

To investigate the clinical factors affecting ACE2 expression in AMs, we collected pulmonary immune cells via BAL from 56 participants who underwent bronchoscopy for clinical purposes. ACE2 expression on AMs was measured using flow cytometry, and the mean fluorescence intensity (MFI) of ACE2 was used as a quantitative indicator to determine the ACE2 expression on AMs. Table 1 shows the clinical characteristics of the participants and their ACE2 expression in AMs. Current smokers exhibited higher ACE2 expression in AMs than non-current smokers (mean (SD) Log_2_ACE2 for current smokers: 12.29 (1.01), for non-current smokers: 11.63 (1.20), P = 0.037). There was no statistically significant difference in ACE2 expression according to age > 65, male sex, overweight, COPD, lung cancer, cardiovascular disease (CVD), diabetes mellitus (DM), or use of renin–angiotensin–aldosterone system (RAAS) inhibitors. The ACE2 expression in AMs was positively correlated with smoking pack-years (r = 0.347, P = 0.038), but not with age, BMI, FEV1, and FEV1/FVC (Fig. 3). Univariate and multivariate linear regression models were used to further investigate the relationship between ACE2 expression of AM and smoking. The results showed that current smokers were associated with increased ACE2 expression in both univariate (β-coefficient: 0.660, 95% CI 0.042–1.279, P = 0.037) and multivariate (β-coefficient: 0.791, 95% CI 0.019–1.562, P = 0.045) models (Table 2). Therefore, these analyses suggest that current cigarette smoking is associated with increased ACE2 expression in AMs.

**Table 1:** Characteristics of participants (N = 56) and the comparison of ACE2 expression of AMs between subgroups.

Characteristic	Number (%)	Mean (SD) ACE2^a^	P-value^b^
Age (years)			
> 65	25 (44.6)	12.04 (1.25)	0.387
≤ 65	31 (55.4)	11.77 (1.10)	
Sex			
Male	41 (73.2)	11.94 (1.15)	0.580
Female	15 (26.8)	11.74 (1.22)	
Overweight			
BMI ≥ 24	25 (44.6)	11.71 (1.27)	0.310
BMI < 24	31 (55.4)	12.03 (1.07)	
Smoking status			
Current smoker	22 (39.3)	12.29 (1.01)	0.037
Not current smoker	34 (60.7)	11.63 (1.20)	
COPD			
Yes	22 (39.3)	12.18 (1.22)	0.126
No	34 (60.7)	11.70 (1.10)	
Lung cancer			
Yes	30 (53.6)	11.84 (1.20)	0.757
No	26 (46.4)	11.94 (1.14)	
CVD			
Yes	21 (37.5)	11.71 (0.86)	0.333
No	35 (62.5)	11.99 (1.31)	
DM			
Yes	10 (17.9)	11.74 (1.11)	0.657
No	46 (82.1)	11.92 (1.18)	
RAAS inhibitor use			
Yes	5 (8.9)	12.10 (1.40)	0.673
No	51 (90.1)	11.87 (1.15)	

*ACE2* angiotensin converting enzyme 2, *AM* alveolar macrophage, *BMI* body mass index, *COPD* chronic obstructive pulmonary disease, *CVD* Cardiovascular disease, *DM* diabetes mellitus, *RAAS* Renin–angiotensin–aldosterone.

^a^ACE2 of AMs were measured by flow cytometry, and the mean fluorescence index value was transformed by log_2_.

^b^Calculated by Student’s *t* test.

**Table 2:** Univariate and multivariate linear regression analyses for ACE2 expression of AMs.

Covariables^b^	Univariable			Multivariable^a^		
	β-coefficient	95% CI	P-value	β-coefficient	95% CI	P-value
Age (years)	− 0.001	− 0.027–0.024	0.899	0.012	− 0.019–0.044	0.435
BMI	− 0.059	− 0.138–0.020	0.141	− 0.028	− 0.122–0.065	0.543
Male	0.197	− 0.511–0.906	0.579	− 0.447	− 1.454–0.560	0.376
Current smoker	0.660	0.042–1.279	0.037	0.791	0.019–1.562	0.045
COPD	0.489	− 0.141–1.119	0.126	0.543	− 0.366–1.451	0.235
Lung cancer	− 0.098	− 0.728–0.533	0.757	− 0.628	− 1.391–0.136	0.105
CVD	− 0.293	− 1.026–0.440	0.426	− 0.304	− 1.032–0.425	0.321
DM	− 0.182	− 1.002–0.638	0.657	0.043	− 0.826–0.912	0.921
RAAS inhibitor	0.233	− 0.869–1.334	0.673	0.720	− 0.487–1.928	0.236

*ACE2* angiotensin converting enzyme 2, *AM* alveolar macrophage, *BMI* body mass index, *COPD* chronic obstructive pulmonary disease, *CVD* cardiovascular disorder, *DM* diabetes mellitus, *RAAS* Renin–angiotensin–aldosterone.

^a^Adjusted age, BMI, male, current smoker, COPD, lung cancer, hypertension, DM, RAAS inhibitor.

^b^References for covariables: male (male versus female), current smoker (current smoker vs non-current smoker), COPD (yes versus no), lung cancer (yes versus no), CVD (yes versus no), DM (yes versus no), RAAS inhibitor (use versus no use). Age and BMI were calculated as continuous variables.

### Cigarette smoking induces ACE2 expression in AMs by increasing the production of ROS

**Figure 4:**
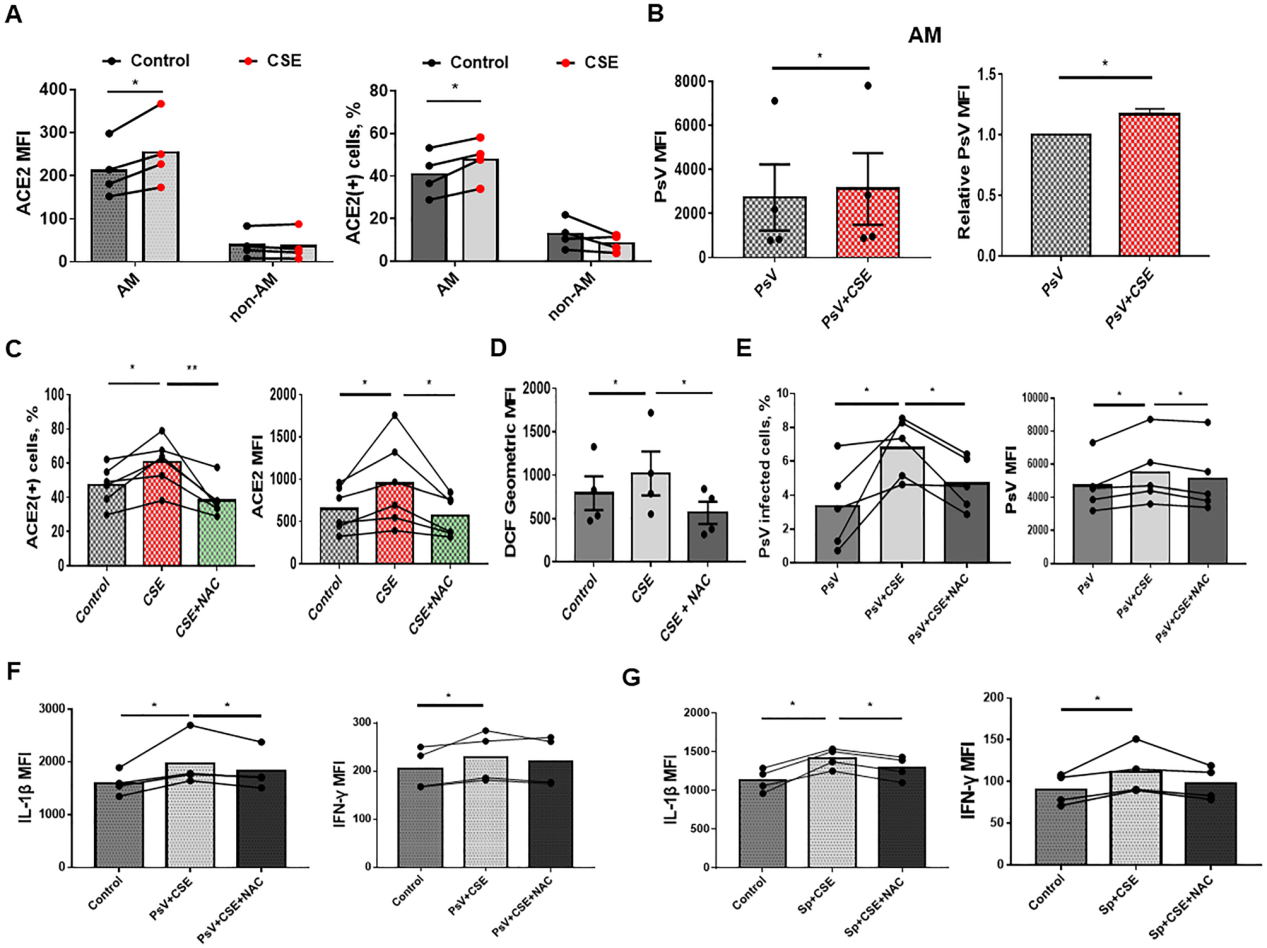
The effect of NAC on CSE-induced ACE2 expression, ROS production, and susceptibility to CoV-2 PsV in AMs. (**A**) The comparison of ACE2 expression and percentage of ACE2-expressing cells between AM and non-AM immune cells before and after treatment with CSE. BAL-derived cells of non-smokers were treated with 1% CSE for 24 h. The ACE2 expression were measured by flow cytometry. (**B**) The comparison of CoV-2 PsV infection between CSE-treated AMs and the control group. BAL-derived AMs were treated with CSE for 24 h and then incubated with CoV-2 PsV for 72 h. (**C**,**D**) Comparison of ACE2 expression and percentage of ACE2-expressing AM **(C)** and ROS production (**D**) between AMs treated with 1% CSE alone, 1% CSE and 10 mM NAC, or the control group. The BAL-derived AMs were treated with NAC and/or CSE for 24 h before measuring ACE2 expression, and 30 min before measuring ROS production. (**E**) The comparison of susceptibility to CoV-2 PsV between AMs treated with CSE alone, CSE and NAC, or the control group. After 24 h of 10 mM NAC and/or 1% CSE treatment, the AMs were incubated with CoV-2 PsV for 72 h, and CoV-2 PsV was measured using flow cytometry. (**F**,**G**) The comparison of cytokine production between AMs treated with 1% CSE alone and 1% CSE and 10 mM NAC after CoV-2 PsV inoculation for 72 h (**F**) or CoV-2 Sp for 24 h (**G**). Intracellular cytokines were measured using flow cytometry. The columns and error bars represent the means and SEMs of experiments. The statistically significant differences between groups are indicated with * and ** (*P < 0.05, **P < 0.01, paired *t* test).

We confirmed the effect of cigarette smoking on ACE2 expression in AMs by treating BAL-derived cells from never-smokers with cigarette smoke extract (CSE) and measuring ACE2 expression using flow cytometry. Both the ACE2 MFI and proportion of ACE2-positive AMs significantly increased after CSE treatment. In contrast, there was no significant change in the ACE2 MFI and proportion of ACE2-positive non-AM pulmonary immune cells. (Fig. 4A). CSE increase type I inflammatory cytokines production but did not lead to an increase in type 2 inflammatory cytokine production (Supplementary Fig. 1), which is consistent with previous studies^23^. To investigate the effect of cigarette smoking on the susceptibility of AMs to SARS-CoV-2, we co-cultured AMs with CoV-2 PsV, and observed that the CSE-treated AMs had more CoV–2 PsV infection than the control group** (**Fig. 4B). N-acetylcysteine (NAC), an adjuvant treatment for COVID–19^24,25^, was used to treat the AMs after CSE exposure. We found that NAC reversed the increase of ACE2 expression and intracellular ROS caused by CSE (Fig. 4C,D). Additionally, NAC treatment reduced SARS-CoV-2 infection in CSE-treated AMs (Fig. 4E). NAC also decreased CoV-2 PsV and CoV-2 Sp- induced IL-1β in CSE-treated AMs. (Fig. 4F,G). Without CSE stimulation, NAC reduced the CoV-2 PsV infection in AMs, as well as decreased CoV-2 PsV and CoV-2 Sp-induced cytokines in the AMs (Supplementary Fig. 2).

**Figure 5:**
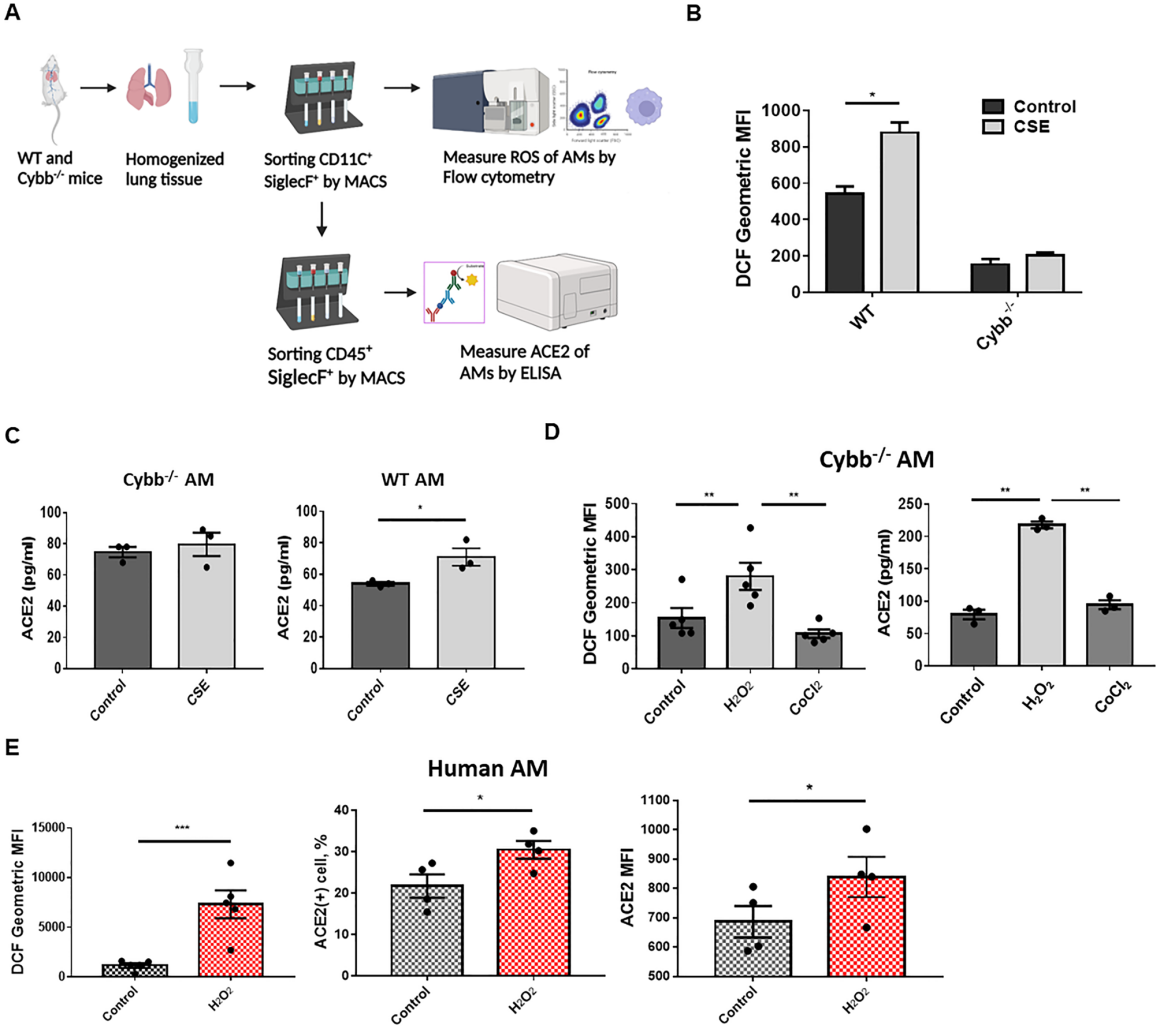
The effect of ROS on ACE2 expression in AMs. (**A**) To estimate ROS production of AMs, CD11c^+^Siglec-F^+^ cells were isolated from lung homogenates of wild-type and Cybb^–/–^ mice using MASC. The ROS production in AMs (CD11c^+^CD45^+^Siglec-F^+^) was then measured using flow cytometry. To estimate ACE2 expression, the second MASC was performed to isolate AMs (CD11c^+^CD45^+^Siglec-F^+^ cells) from CD11c^+^Siglec-F^+^ cells, and ELISA was used to measure ACE2 expression in AMs. (**B**,**C**) The comparison of intracellular ROS (n = 4) (**B**) and ACE2 expression (n = 3) (**C**) between CSE-treated wild type and Cybb^–/–^ AMs. The AMs were treated with 1% CSE for 30 min before measuring ROS production and 24 h before measuring ACE2 expression. Pooled AMs from 5 mice were used for each independent experiment. (**D**) The comparison of intracellular ROS and ACE2 expression between wild-type and Cybb^–/–^ AMs after treatment of 1 mM H_2_O_2_ for 15 min or 10 mM CoCl_2_ for 60 min. Pooled AMs of 5 mice were used for each independent experiment. (**E**) The comparison of intracellular ROS and ACE2 expression of AMs between H_2_O_2_-treated and control group. The dose and duration of H_2_O_2_ treatment were the same as in Fig. 5D. The columns and error bars represent the means and SEMs of experiments. The statistically significant differences between groups are indicated with * and ** (*P < 0.05, **P < 0.01, paired *t* test).

As the trends in ROS production and ACE2 expression in AMs showed similarities in response to CSE and NAC, we hypothesized that CSE increases ACE2 expression by elevating the intracellular ROS of AMs. To test this hypothesis, we isolated AMs from Cybb^–/–^ and wild type mice using the magnetic-activated cell sorting (MACS) technique and measured intracellular ROS production and ACE2 expression using flow cytometry and ELISA, respectively. (Fig. 5A). Compared to AMs from wild type mice, CSE did not significantly increase ROS production (Fig. 5B) and ACE2 expression in AMs from Cybb^–/–^ mice (Fig. 5C). However, exogenous ROS provided by H_2_O_2_ could increase ROS production and ACE2 expression in AMs from Cybb^–/–^ mice. CoCl2, a ROS-independent HIF-1α stabilizer, did not increase ROS production and ACE2 expression in AMs from Cybb^–/–^ mice (Fig. 5D). Administration of H_2_O_2_ also increased ROS and ACE2 expression in primary-cultured human AMs (Fig. 5E). These results suggest that cigarette smoking increases ACE2 expression in AMs by inducing ROS production, independent of HIF-1α stabilization, thus further increasing susceptibility to SARS-CoV-2.

## Discussion

This study investigated the clinical factors that affect the expression of ACE2 in immune cells in the lungs. We identified AMs as the major immune cells expressing ACE2 in the human lung, which were found to be more immunogenic to CoV-2 Sp and more susceptible to CoV-2 PsV infection. In a multivariate analysis of BAL-derived cells from 56 clinical patients, current smoking was identified as an independent risk factor for increased ACE2 expression in AMs. Our in vitro studies demonstrated that CSE increased the susceptibility of AMs to CoV-2 PsV, induced ACE2 expression in AMs by increasing intracellular ROS, and that NAC reduced ACE2 expression in AMs by suppressing ROS. This study established a causal relationship between cigarette smoking, ACE2 expression, SARS-CoV-2 infection in AMs. Another strength of this study is the combination of clinical and in vitro research to explore the mechanism of smoking-induced ACE2 expression in AMs.

This study demonstrated that AMs with high ACE2 expression were more susceptible to CoV-2 PsV and produced significantly higher levels of cytokines when treated with CoV-2 Sp and CoV-2 PsV. AMs play a crucial role in coordinating inflammation, damage, and repair processes, which determine the various pathological stages of ARDS^17^. Dysregulated cytokine production and resulting cytokine storms, which induce ARDS^26^, are widely reported as leading causes of death among COVID-19 patients^27^. Moreover, COVID-19 patients with cytokine storms have a poor prognosis^28^. Reducing cytokine storms is crucial for the treatment of COVID-19 patients. Immunomodulators such as the anti-IL-6 antibody and JAK inhibitors have been shown to improve the survival of hospitalized COVID-19 patients^29,30^. Our study suggested that decreased ACE2 expression can reduce the susceptibility of AMs to CoV-2 PsV and subsequently decrease cytokine production. However, further clinical studies are required to investigate the clinical effectiveness of this approach.

We found that CSE-treated AM are more susceptible to CoV-2 PsV infection. Initially, during the COVID-19 outbreak, several studies reported a relatively low prevalence of smoking among COVID-19 infected patients^31–33^. However, later studies with a larger sample size have shown that current smoking is independently associated with an increased risk of self-reported confirmed COVID-19 infection^34,35^. From a pathophysiological perspective, cigarette smoking disrupts the airway epithelial barrier and impairs ciliary oscillations, leading to the accumulation of excessive mucus secretion in the airway. This environment favors pathogen colonization and reproduction^36^. Long-term cigarette smoking can also impair innate immunity, inhibit dendritic cell maturation and IFN-α production^37^, and hinder macrophage phagocytosis, which leads to the rapid transmission of pathogens^38^. Previous studies have shown that smokers have an increased risk of viral infections, including Middle East respiratory syndrome coronavirus and influenza^39,40^. Consistent with these findings, our results suggest that smokers are more likely to be infected with SARS-CoV-2.

Cigarette smoke is a complex mixture of over 5000 chemical compounds^41^. Smoking induces sustained low-intensity inflammation by increasing cellular oxidative stress^42^. Previous studies have shown that smoking increases ACE2 expression in secretory cells, bronchial and alveolar epithelia^43–45^. Similarly, our study showed that CSE increased ROS production and ACE2 expression in AMs, from both humans and mice in a similar trend. Furthermore, CSE did not significantly increase ACE2 expression in AMs of Cybb^–/–^ mice, which lack the enzyme responsible for generating endogenous ROS. In contrast, exogenous ROS provided by H_2_O_2_ increased ACE2 expression in AMs of Cybb^–/–^ mice. These results suggest that CSE increases ACE2 expression in AMs mainly by increasing endogenous ROS production.

The role of HIF-1α in ACE2 expression is controversial. Liu et al. reported that smoking increases HIF-1α in both mouse and human bronchioles, which transcriptionally upregulates ACE2 expression by binding to its promoter^44^. However, Zhang et al. have found that ACE2 mRNA and protein increase early in hypoxia but decrease to baseline levels after accumulation of HIF-1α, induced by ACE1-catalyzed angiotensin II, using pulmonary artery smooth muscle cell lines^46^. In this study, we found that CoCl_2_, a HIF-1α stabilizer, did not increase ACE2 expression in AMs of Cybb^–/–^ mice. However, the baseline ACE2 level of Cybb^–/–^ mice was not inferior to wild-type mice, suggesting that ACE2 production does not rely exclusively on intracellular ROS.

In this study, we found that NAC could decrease ACE2 expression in AMs. NAC has been used as an adjuvant treatment for COVID-19^24,25^, but its treatment effect has not yet been proven by clinical studies^47,48^. Our results showed that NAC decreased the susceptibility of CoV-2 PsV and reduced the inflammatory cytokine induced by CoV-2 PsV and CoV-2 Sp through downregulation of ACE2 expression in CSE-treated AMs, suggesting a potential preventive effect against severe respiratory complications associated with SARS-CoV-2 infection. Nevertheless, further clinical studies are required to investigate the effectiveness of NAC as a treatment for COVID-19.

An interesting finding of this study is that cigarette smoke did not increase ACE2 expression in non-AM pulmonary immune cells. Previous studies have indicated that besides macrophages, other pulmonary immune cells rarely express ACE2^49^. In addition, the effect of cigarette smoke on ACE2 expression may differ between cell types. Tanimoto et al. have reported that CSE inhibited ACE2 expression in HepG2 cells by upregulating aryl hydrocarbon receptor-targeting genes^50^. Muus et al. showed that cigarette smoke did not increase ACE2 expression on alveolar type 2 cells^8^. Since we did not classify the non-AM pulmonary immune cells in this study, further research is needed to investigate the effect of CSE on ACE2 expression in other types of pulmonary immune cells.

This study had some limitations. First, for safety and infection control reasons, we used CoV-2 PsV instead of true SARS-CoV-2 in our cell study. Lentivirus-based SARS-CoV-2 spike PsV has been widely used to study the entrance and infection of SARS-CoV-2^51,52^. Spike protein has also been used to survey the inflammatory responses in human macrophages^53^. Moreover, our study results are consistent with previous studies that used the true virus^6,54^. Therefore, the findings using CoV-2 PsV and CoV-2 Sp are reliable. Second, the AMs used in this study were obtained from a Taiwanese population, and the susceptibility to smoking may vary by race^55^. However, due to geographical limitations, we could not obtain clinical specimens from Westerners and other races.

## Conclusions

The results of this study suggest that cigarette smoking increases the susceptibility of AMs to SARS-CoV-2 by upregulating ACE2 expression through ROS-related mechanisms, and that NAC can reduce ACE2 expression in AMs by suppressing ROS. These findings emphasize the importance of smoking cessation in preventing severe pulmonary infections in COVID-19 patients, and further research is needed to investigate the efficacy of NAC in preventing severe complications of COVID-19.

## Methods

### Study design and participants enrollment

To obtain the AMs from clinical patients, we prospectively enrolled participants who receiving scheduled bronchoscopy for clinical purposes at the National Cheng Kung University Hospital (NCKUH) between June 2020 and January 2022. The Institutional Review Board of NCKUH approved this study (B-ER-109-016) prior to commencement. Written informed consent was obtained from all subjects. Exclusion criteria were as follows: (1) age < 20 years; (2) pulmonary infection or exacerbation of inflammatory airway disease within one month; (3) had diffuse pulmonary lesion or interstitial lung disease; (4) received inhaled or systemic immunosuppressive therapy; (5) had been infected with COVID-19; (6) received NAC. Baseline information of the participants, including demographics, smoking status, comorbidities, concurrently used medication, pulmonary function test, and chest computed tomography (CT) images were collected from electronic medical records in the NCKUH database. Participants who had persistently stopped smoking for more than one month were defined as ex-smokers. Participants were defined as having COPD if the forced expiratory volume in one second (FEV1) to forced vital capacity (FVC) ratio was < 70%. For participants who had not received pulmonary function test, they were defined as COPD if they had been smoking for more than 10 pack-years in combination with diffuse pulmonary emphysema on chest CT^43^.

### Collection of pulmonary immune cells

We used BAL to collect pulmonary immune cells from the participants. The procedures were performed in accordance with the recommended practice guidelines^56^. Briefly, BAL is performed with the fiberoptic bronchoscope after wedging in the targeted segmental bronchus. For participants with unilateral lung lesions, BAL was performed in the unaffected side of lung. The first instilled aliquot of 25 mL isotonic sodium chloride solution was discarded to avoid contamination of bronchial secretions. The following instilled aliquots were withdrawn, and the total installed and retrieved volume ranged from 100–300 mL to 30–50 ml, respectively. The retrieved BAL fluid immediately stored in 4 degrees Celsius refrigerator and filtered using a 70 μm strainer and centrifuged at 1500 RPM for 10 min later. The pellets of BAL cells were washed and re-expanded using PBS for flow cytometry or culture.

### Classification of pulmonary immune cells, and quantification of ACE2 and cytokine production

We used flow cytometry (FACS Canto II (BD Biosciences)) to classify pulmonary immune cells from BAL and quantify the ACE2 expression. Antibodies were purchased from BD Biosciences (NJ, USA), and Biolegend (CA, USA). To block non-specific antibody binding of immune cells, BAL cells were inoculated in staining buffer with Human BD Fc Block for 30 min. Then, the following antibodies were used: FITC-conjugated anti-CD45 (2D1), PE-conjugated anti-CD11b (ICRF44), PerCP-conjugated anti-CD15 (W6D3), APC-cy7-conjugated anti-CD16 (3G8), BV510-conjugated anti-CD169 (3G8), BB700-conjugated anti-CD206 (19.2), PE-conjugated anti-IL-1β (AS10), APC-conjugated anti-IL-10 (JES3-19F1), PE-cy7-conjugated anti-IFN-γ (B27), PE-conjugated anti-ACE1 (BB9) and AF647-conjugated anti-ACE2(A20069I). AM, non-AM macrophages, neutrophil and lymphocytes were defined as follow: AM (CD11b^+^CD16^+^CD45^+^CD169^+^ CD206^+^), non-AM macrophages (CD11b^+^CD15^–^CD16^+^CD45^+^CD169^–^CD206^+^), Neutrophil (CD11b^+^CD15^+^CD16^+^CD45^+^CD169^–^) and lymphocytes (CD45^+^CD11b^–^)^22,57^. Quantitative flow cytometry was performed to quantify ACE2, IL-1β, IL-6, IL-10, and IFN-γ in cells by calculating the MFI of each substance. MFI can be used as a quantitative indicator for the study materials^58^. Fluorochrome minus one control with corresponding isotype control antibodies (IgG1-PE, IgG1-FITC, IgG1-PE-cy7, IgG1-APC-cy7, IgG1-BB700, IgG1-PerCP, IgG1-BV510, IgG2A-AF647, IgG2A-APC BD Biosciences) was performed to eliminate the interference of background fluorescence. The flow cytometry data were acquired on a FACSCanto II instrument (BD Biosciences) and analyzed using FlowJo software (TreeStar).

### SARS–CoV–2 spike protein stimulation and pseudovirus infection assay

SARS-CoV-2 spike protein (CoV-2 Sp; Leadgene Biomedical, Inc, Taiwan) and lentivirus–based spike protein expressing pseudovirus (CoV-2 PsV; ACE Biolabs CO., Taiwan) were used to test the susceptibility of AMs to SARS-CoV-2 infection. First, ex-vivo human AMs were inoculated in 96-well cell culture plates at a volume of 4 × 10^4^ cells/well. For the CoV-2 Sp assay, 5 μg/ml of CoV-2 Sp was placed in each well and inoculated for 24 h. Then, ACE2, IL-1β, IL-6, and IFN-γ expression levels in AMs were measured using flow cytometry. For the pseudovirus infection assay, 6 μL of CoV-2 PsV was added and infected for 72 h. Then, the infection efficiency was determined using green fluorescent's mean fluorescence intensity in flow cytometry.

### CSE preparation

The CSE preparation protocol was adapted from previous study with modification^59^. Briefly, five cigarettes (Marlboro red; tar: 10 mg, nicotine, 0.8 mg; Philip Morris USA Inc) was burned. The smoke was sequentially bobbled into 10 ml vessel containing phosphate-buffered saline using a syringe pump at a flow rate of 300 ml/min. The CSE was sterilized by filtrated via a 0.2-μm Millipore filter before administration. The CSE was prepared freshly and diluted by phosphate-buffered saline to a 1% solution for each utilization. The results of the dose and time response of ACE2 in human AMs to CSE administration, as well as the positive control with metformin^60^, are presented in Supplementary Fig. 3. Based on the test results, we treated AMs with 1% CSE for 24 h in our experiments.

### Measurement of intracellular ROS levels

Ex-vivo AMs were washed with suspension buffer (1X phosphate buffered saline and 2% fetal bovine serum) three times and incubated with 1% CSE and NAC (10 mM; Sigma-Aldrich) at 37 °C for 30 min in the presence of 20 mM DCF-DA (abcam) at 37 °C. After stimulation with DCF-DA for the desired time, the cells were washed and followed by fluorescence at Ex/Em = 485/535 nm and analysis with flow cytometry. Data were analyzed with FlowJo software (TreeStar).

### Preparation of AMs from wild type and Cybb^–/–^ mice

AMs were obtained from C57BL/6 wild type and Cybb^–/–^ mice aged 8–12 weeks. The protocol for lung extraction and digestion was adapted from a previous study^61^. Briefly, the mice’s lungs were extracted under anesthesia induced by an intraperitoneal injection of Zoletil (50 mg/kg body weight) and Rompun (2.332 mg/kg body weight) cocktail. The cardiac ventricles were dissected and flushed with 2 ml of phosphate-buffered saline (PBS) with 0.1% heparin to remove blood in the pulmonary circulation. Then, both lungs were removed separately and minced with scissors into 2–3 mm pieces, which were digested in medium (RPMI 1640, 1% penicillin/streptomycin; Gibco, Waltham, MA, USA) with Liberase thermolysin medium (100 μg/mL; Roche, Basel) and DNase I (1 μg/mL; Sigma, Saint Louis, MO, USA) solution at 37 °C for 30 min in a shaker. After digestion, the fluid was filtered using a 70-μm nylon mesh (BD Biosciences, San Jose, CA, USA) and re-suspended over a 50 ml conical tube. Then, the cell pellets were obtained through centrifugation at 1200 rpm for 10 min at 4 °C. The protocol for isolation and culture of AMs was adapted from previous studies^62,63^. AMs were processed after acquisition using the MACS cell separation (Miltenyi, Auburn, CA, USA) and a CD11C, Siglec-F positive selection on a magnetic column. Cell suspensions were filtered using a 40 μm nylon mesh. Cell viability, CD45, CD11C, and Siglec-F positive cell purity and characterization of surface marker expression were assessed using a flow cytometer. All experiments were conducted according to the National Institute of Health guidelines for animal care and were approved by the Institutional Animal Care and Use Committee (IACUC) at National Cheng Kung University (IACUC Approval Number: 111258). Study details are in accordance with ARRIVE guidelines.

### Measurement of ACE2 expression in mice AMs using ELISA

CD45^+^Siglec-F^+^ cells were selected from the cultured CD11C^+^Siglec-F^+^ population using magnetic–activated cell sorting to enhance the purity of AMs. Then, the AM lysates were harvested with 100 μl of RIPA lysis buffer (Sigma, Saint Louis, MO, USA) with a 50X protease inhibitor mixture. Samples were centrifuged at 12,000 rpm for 20 min at 4 °C to pellet cell debris. ACE2 expression in the whole lung AM culture samples were measured using a duplicate CUSABIO mouse ACE2 enzyme-linked immunosorbent assay (ELISA) Kit (CSB-E17204m, CUSABIO, Houston, TX, USA), according to the manufacturer’s instructions.

### Statistical analysis

In the clinical cohort study, we utilized the MFI of ACE2 as an indicator for ACE2 expression on AMs. The ACE2 MFI was transformed using log_2_ to establish linear data. The Student’s *t* test was used to compare the mean ACE2 between different subgroups. Spearman’s correlation tests were conducted to analyze the correlations between ACE2 and clinical factors. Univariate and multivariate linear regression analyses were performed to investigate the association between ACE2 expression and clinical factors. Age, sex, body mass index (BMI), and other clinical factors, such as smoking status, COPD, lung cancer, DM, CVD, use of RAAS inhibitor, all of which are known to increase ACE2 expression, were entered into the multivariate linear regression model as covariates^43,44,64–66^. Among the covariates, age and BMI were entered into the model as continuous variates, whereas sex (male vs. female), smoking status (active smoker vs. ex– or never–smoker), COPD (with vs. without), lung cancer (with vs. without), and RAAS inhibitor (use vs. no use) were entered as categorical variates. For in vitro laboratory research, the data were expressed as mean and Standard Error of the Mean (SEM). A paired *t* test was performed to compare the ACE2 expression between the controls and investigating samples. Statistical significance was set at two–sided P < 0.05. SAS software (version 9.4, Cary, NC: *SAS* Institute Inc; 2014) and GraphPad Prism 7 (GraphPad Software, San Diego, California USA) were used for statistical analysis.

### Ethics declarations

This study was approved by the Institutional Review Board of National Cheng Kung University Hospital before commencement (B-ER-109-016). Written informed consent was obtained from all subjects. All experiments of animals were conducted according to the National Institute of Health guidelines for animal care and were approved by the Institutional Animal Care and Use Committee (IACUC) at National Cheng Kung University (IACUC Approval Number: 111258). Study details are in accordance with ARRIVE guidelines.

## Supplementary Information

Supplementary Figures.

## Data Availability

All data are available from the corresponding author upon reasonable request.
