# Supplementary material for: Cigarette smoke increases susceptibility of alveolar macrophages to SARS-CoV-2 infection through inducing reactive oxygen species-upregulated angiotensin-converting enzyme 2 expression
Source: Sci Rep. 2023 May 16;13:7894. doi: 10.1038/s41598-023-34785-6 (PMC10185955; doi:10.1038/s41598-023-34785-6)
Supplement: Supplementary file 1 — Supplementary Figures. [file 41598_2023_34785_MOESM1_ESM.docx]

**Supplementary Figure 1**


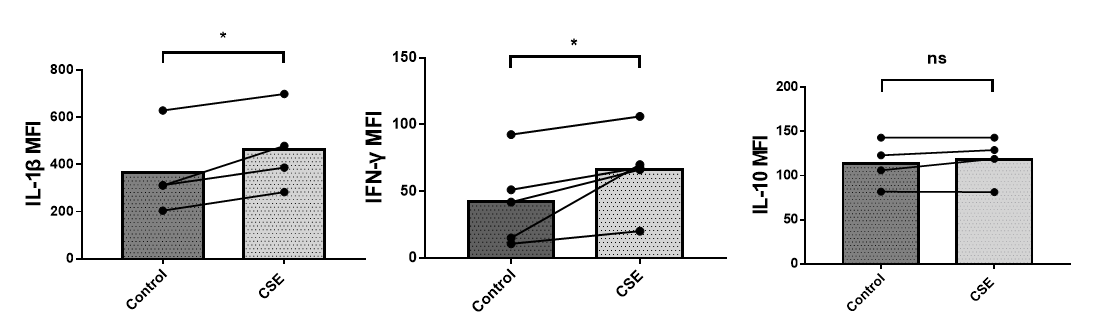


**Supplementary Figure 1.** Type I and type II inflammatory cytokines production of AMs after CSE administration. AM of non-smokers were treated with 1% CSE for 24 h. Cytokines were measured by flow cytometry. The columns represent the means of experiments. * indicated p value < 0.05.

**Supplementary Figure 2**


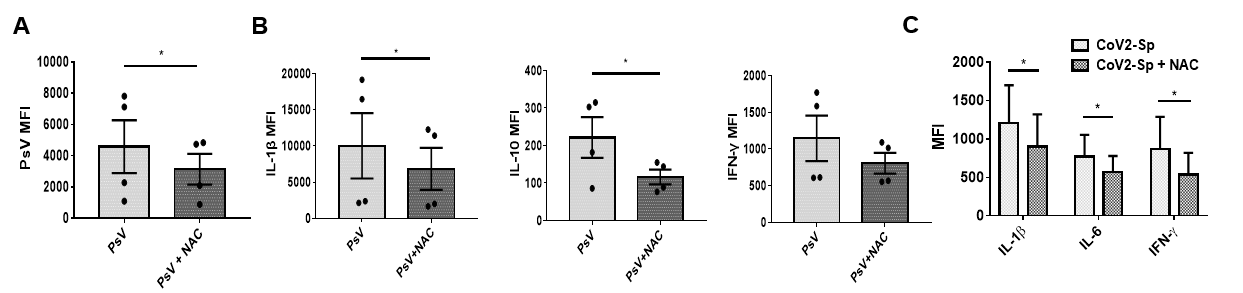


**Supplementary Figure 2.** The comparison of susceptibility to CoV-2 PsV **(A)** and cytokine production **(B)** between NAC-treated AMs and the control group. The BAL-derived AMs were treated with 10 mM NAC for 24 h and then incubated with CoV-2 PsV for 72 h. **(C)** The comparison of IL-1β, IL-6, and IFN-γ levels between NAC- and/or CoV-2 Sp-treated AMs. The BAL-derived AMs were treated with 10 mM NAC and CoV-2 Sp for 24 h before measuring the levels of IL-1β, IL-6, and IFN-γ using flow cytometry. The columns and error bars represent the means and SEMs of experiments. * indicated p value < 0.05.

**Supplementary Figure 3**


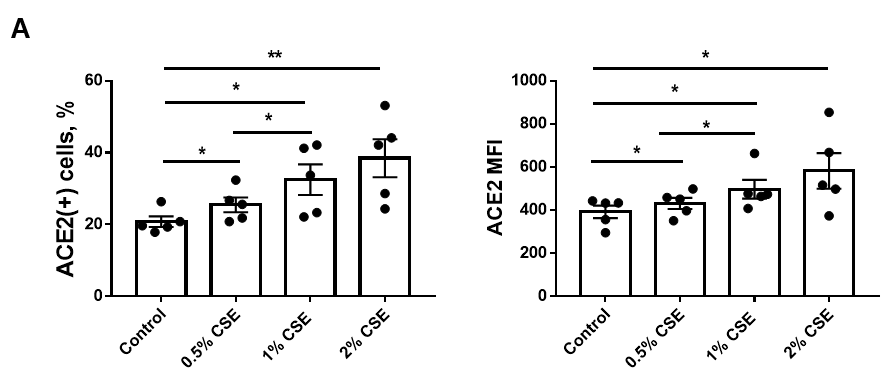


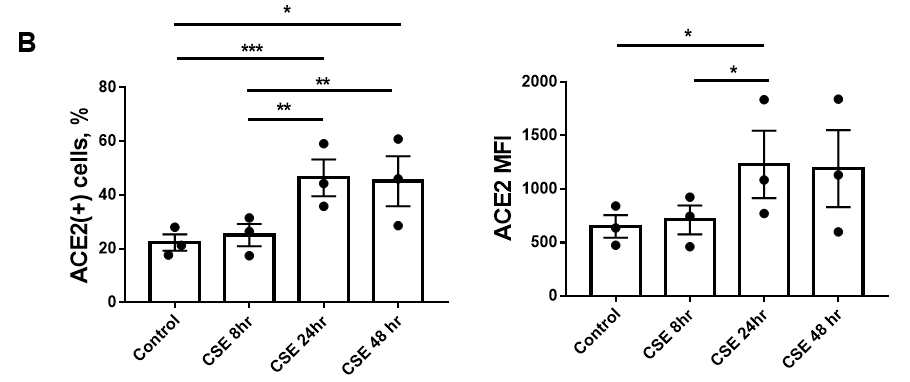


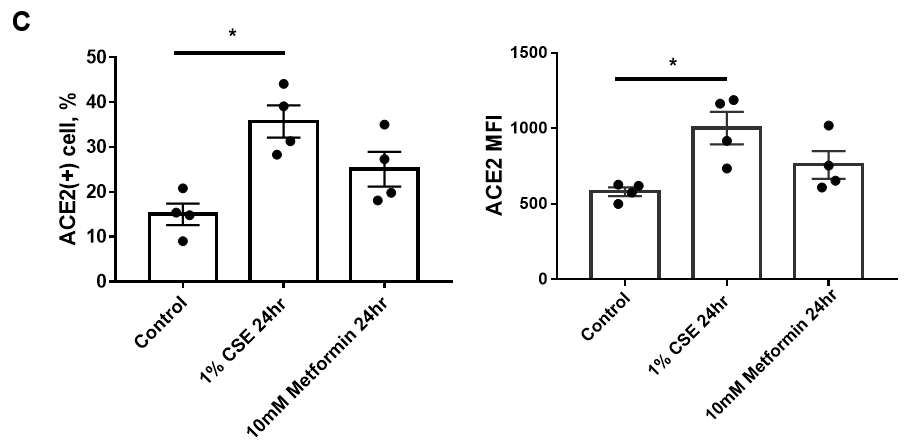


**Supplementary Figure 3.** Dose and time response of ACE2 in human AMs to CSE administration and the positive control of ACE2. (A) Dose response, (B) time response, (C) positive control with 10mM metformin. The ACE2 of AMs were measured by flow cytometry. The columns and error bars represent the means and SEMs of experiments. The statistically significant differences between groups are indicated with *, **, and *** (*p < 0.05, **p < 0.01, ***p < 0.005, paired t test).
